# Workplace violence against healthcare workers during the COVID‐19 pandemic in Sudan: A cross‐sectional study

**DOI:** 10.1002/puh2.31

**Published:** 2022-11-02

**Authors:** Yasir Ahmed Mohammed Elhadi, Hammad Mohamed Hammad Mohamed, Abdelmuniem Ahmed, Islam Hamza Haroun, Mohamed Hassan Hag, Ehssan Farouk, Moaaz Almadani, Alanood Elnaeem Mohamed, Mohammed Fathelrahman Adam, Osman S. Abdelhamed, Elhadi Basheer Salih, Sahar Khalid Mohamed, Mohammed Osman Omer Sanosi

**Affiliations:** ^1^ Department of Public Health Medical Research Office, Sudanese Medical Research Association Khartoum Sudan; ^2^ Al‐Mana General Hospital Al‐Jubail Saudi Arabia; ^3^ Physiology Department Faculty of Medicine University of Gezira Wad Medani Sudan; ^4^ Department of Anatomy Faculty of Medicine and Medical Sciences Omdurman Islamic University Omdurman Sudan; ^5^ Federal Ministry of Health Khartoum Sudan; ^6^ Faculty of Pharmacy University of Science and Technology Khartoum Sudan; ^7^ Faculty of Medicine Ahfad University for Women Omdurman Sudan; ^8^ Faculty of Public Health University of Shendi Shendi Sudan

**Keywords:** COVID‐19, healthcare workers, Sudan, workplace violence

## Abstract

**Background:**

Workplace violence (WPV) against healthcare workers (HCWs) is a growing global issue. During the coronavirus diseases‐2019 (COVID‐19) pandemic, violent attacks on HCWs have been documented worldwide. This study aimed to investigate the magnitude and pattern of WPV among HCWs in Sudan during the COVID‐19 pandemic.

**Methods:**

A web‐based cross‐sectional study of WPV was conducted among registered medical and health workers (pharmacists, physicians, dentists, nurses, laboratory technicians, and administrative and paramedical staff) during the COVID‐19 pandemic in Sudan. Data were collected from August to December 2021 using a self‐administered questionnaire distributed through social media platforms.

**Results:**

A total of 792 HCWs returned the online questionnaire. The mean age was 33.5 ± 8.6 years, where more than half were females (54.9%) and working during the day shift (58.8%). During the COVID‐19 pandemic, three out of every four participants (78.3%) reported experiencing violence, with 65.8 % experiencing it more than three times. The common types of violence experienced were verbal (91.6%), physical (50.0%), and sexual abuse (11.0%). The emergency department reported the highest number of violent incidents (46.9%). Half of these violent events were not reported (50.3%), primarily due to a lack of a reporting system. The demographic factors that were significantly associated with exposure to violence were participants’ occupation (*p* < 0.001), age (*p* = 0.001), marital status (*p* = 0.002), and years of working experience (*p* = 0.020).

**Conclusion:**

WPV was rampant among the HCWs in Sudan during the COVID‐19 pandemic. The current findings are presented to draw the attention of policy leaders and stakeholders in Sudan to this alarming problem prompting the pressing need for policy and system interventions.

## INTRODUCTION

Workplace violence (WPV) against healthcare workers (HCWs) is a growing global issue in developing and developed countries. In low and middle‐income countries (LMICs), violent attacks are rarely addressed due to the lack of effective governmental laws and policies protecting the HCWs [1]. The National Institute for Occupational Safety and Health defines WPV as “any violent action, including physical assaults or threats of assaults, which is directed toward persons at work or on duty” [2, 3]. These violent actions can be physical, verbal, or sexual, with verbal assaults being the most common type encountered [4]. In rare cases, violence can take the form of grievous harm. It can happen in any department in the hospital and at any time with no regard or respect by the perpetrator to the place and the sick patients around [2]. WPV can occur for various reasons, including communication breakdown, unreasonable expectations, and perceived substandard care. Other reported reasons include the perceived long waiting time and the failure to meet patient and family expectations [5, 6].

WPV is a serious and challenging issue in healthcare settings, especially in the situation of the coronavirus disease‐2019 (COVID‐19) pandemic [7, 8]. The International Committee of the Red Cross (ICRC) reported 611 incidents of COVID‐19–related physical or verbal assaults, threats, or discrimination directed against HCWs, patients, and medical facilities in more than 40 countries during the first 6 months of the pandemic [9]. In a survey of violence in the United States in 2020, 20% of participants reported increasing on‐the‐job violence, which they attributed to COVID‐19–related staffing shortages, changes in their patient population, and visitor restrictions [10]. During the pandemic, there has been an increase in reports of WPV attacks against HCWs in LMICs. Mistrust in HCWs, belief in conspiracy theories, limited capacity for admission of COVID‐19 patients in hospitals, hospital COVID‐19 policies, and blaming the HCWs for COVID‐19 deaths were the most frequently cited reasons [11].

Many HCWs treating COVID‐19 in Sudan have reported physical and verbal violence, damage to wards and machines, and family members forcibly entering doctors’ rest lounges [12]. This has resulted in multiple strikes and demonstrations by doctors to criminalize the attacks on HCWs. A new law was approved by the transitional government in Sudan on May 25th, 2020, that stipulates “verbal or physical abuse of medical professionals, disrupting work or destroying assets in medical facilities, and publishing incorrect information that affects the performance of medical personnel can be punishable with imprisonment for up to 10 years” [13]. WPV to the HCWs has an impact on their psychological state and work performance and results in demotivation, poor job satisfaction, and early physician burnout [14, 15, 16]. Furthermore, it has been linked to a higher risk of suffering post‐traumatic stress disorder, anxiety, and depression [17, 18]. This unfortunate incident also extends to influence career choices and decisions among medical students [19].

Considerable evidence documented an increase in WPV against HCWs amidst COVID‐19.  For instance, a study in Iraq found that rates of violence against HCWs have increased since the pandemic began [20]. Similarly, in Jordan, there was a significant rise in violence and intimidation against HCWs and medical infrastructure [5]. Also in Egypt, a study showed a high magnitude of violence against HCWs during the pandemic [21]. Previous studies of WPV in Sudan have focused on physicians and were conducted mainly in Khartoum state [14, 22]. However, there is a paucity of information regarding the extent of WPV and its associated factors among other HCWs in different parts of Sudan during the pandemic. The current study was therefore designed to investigate the extent and pattern of WPV among HCWs during the COVID‐19 pandemic in Sudan.

## METHODS

### Study design and sample population

This was a cross‐sectional study reported using the Strengthening the Reporting of Observational Studies in Epidemiology (STROBE) statement [23]. The target population was registered medical and other health staff in Sudanese hospitals and health clinics. Pharmacists, physicians, dentists, nurses, laboratory technicians, and administrative and paramedical personnel aged 18 and above who worked in a hospital or health center during the data collection period and who had access to the internet were eligible to participate. The sample estimation was based on the total number of health workers, an alpha error of 0.05, and a margin of error of 5%, assuming a 50% predicted magnitude of WPV. The minimum required sample size was 381 participants. The calculation was done through Epi info‐7 software.

### Study variables, survey tool, and data collection

The dependent variable considered in this study was suffering from WPV. The question used to estimate this was “Have you encountered WPV during the COVID‐19 pandemic?” with a (yes/no) answer. We did not utilize standard measurements of WPV, which are based on long/multiple questions of WPV experience, [24, 25] to avoid having a lengthy questionnaire. While age, gender, employment status, marital status, years of job experience, and time‐shift work (either day or night) were the independent variables in this study. Other parameters assessed using closed‐ended questions include frequency of WPV (once, twice, or three times), type (verbal, physical, sexual, or other), anticipated stressors, and reporting of violence experienced.

An online, self‐administered and structured questionnaire was used for data collection with the survey questions adapted from previous studies on WPV [14, 26]. The questionnaire consisted of two parts; the first part explored the sociodemographic and work‐related characteristics of respondents and whether they experienced WPV during the COVID‐19 pandemic or not. The second part comprised further questions to those polled, it was about violence patterns experienced (frequency, type, reasons, and reporting of attacks). The link to the final questionnaire was distributed to participants using Google forms through electronic mail addresses and virtual groups on social media platforms, including Facebook, WhatsApp, Twitter, and Telegram. Data were collected from August to December 2021. To account for sample selection bias, we distributed the electronic survey using verified electronic mail addresses of HCWs in Sudan. In addition, the sample size was increased by 100% to account for future need for stratification. Moreover, it was not permitted to submit more than one survey.

### Data analysis

Data analysis was done using IBM SPSS software package version 20.0. (Armonk, NY: IBM Corp). The Kolmogorov–Smirnov was used to verify the normality of the distribution of variables. Descriptive statistics were produced. The bivariate analysis was conducted to examine factors associated with suffering WPV. The significance of the obtained results was determined at a *p* < 0.05.

### Ethical considerations

This study has been approved by the Health Sector Ethical Review Committee, Faculty of Medicine, University of Gezira, Sudan. An information sheet outlining the research purpose was sent before the questionnaire, followed by an invitation to participate and informed consent.

## RESULTS

A total of 792 HCWs responded and returned the online questionnaire. The sample size more than doubled based on the enthusiastic response to the survey. These respondents are part of the human resources of Sudan embedded in a health system based on the district health system approach, with an estimated number of 1.2 hospitals and 13.5 primary health clinics serving 100,000 residents. According to the 2019 statistical report of the Federal Ministry of Health in Sudan, the total number of registered medical and other health workers is 49,354, with 5.6 physicians and 47.6 nurses and midwives per 10,000 population.

The respondents have a mean age of 33.5 ± 8.6 years. Over half of the respondents were females (54.9%) and working during the day shift (58.8%). Many of the participants were physicians (43.9%), single (49.2%), and with < 5 years of experience (48.2%) (Table 1).

**TABLE 1:** Sociodemographic and work‐related characteristics among HCWs surveyed for WPV during the COVID‐19 pandemic in Sudan, 2021 (*N* = 792)

Sociodemographic and work‐related characteristics	*n*	(%)
Age (years)		
<30	356	(45.0)
30– < 40	241	(30.4)
≥40	195	(24.6)
Gender		
Male	357	(45.1)
Female	435	(54.9)
Marital status		
Married	366	(46.2)
Single	390	(49.2)
Divorced/Widow	36	(4.6)
Occupation		
Physician	348	(43.9)
Nurse	159	(20.1)
Dentist	85	(10.7)
Pharmacist	124	(15.7)
Laboratory technician	58	(7.3)
Others	18	(2.3)
Work experience (years)		
<5	382	(48.2)
5–10	207	(26.1)
>10	203	(25.7)
Shift time worked		
Day shift	466	(58.8)
Night shift	326	(41.2)

**FIGURE 1:**
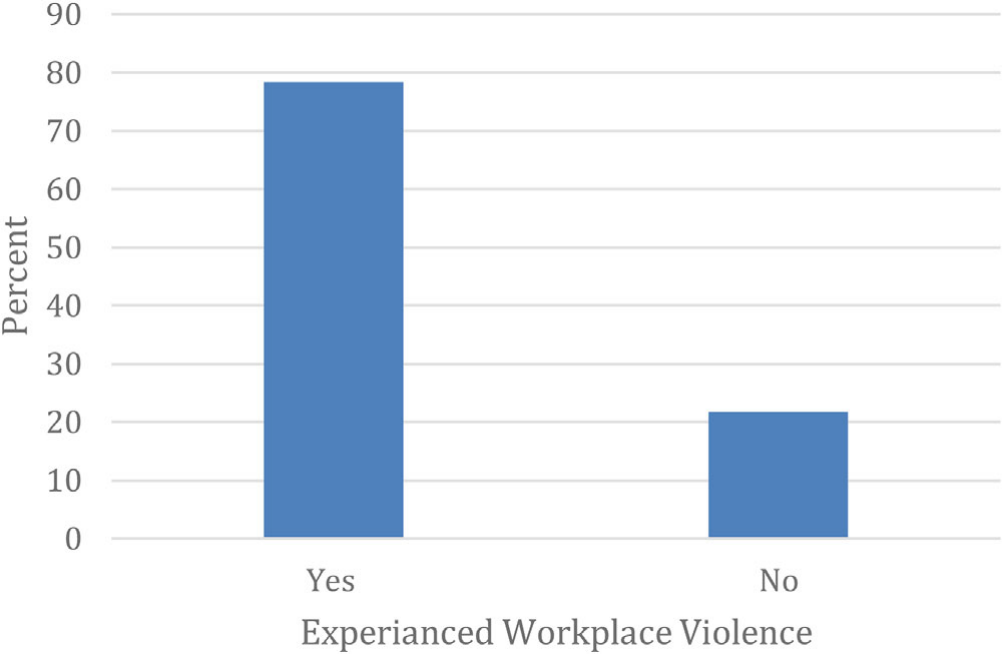
The magnitude of WPV among HCWs during the COVID‐19 pandemic in Sudan, 2021 (*n* = 792)

The magnitude of WPV is shown in Figure 1. Three in every four participants (78.3%) reported experiencing violence during the COVID‐19 pandemic. The frequency and pattern of violence against HCWs are presented in Table 2. Nearly two‐thirds (65.8%) reported experiencing violence more than three times. The most common type was verbal (swearing or screaming) violence, reported by 91.6 % of those polled, while half of the respondents suffered physical violence (50%). The emergency department was the primary location of 46.9 % of the violent incidents. In most incidents (87.9%), patients’ families were the offenders, and the most perceived triggers for violence were lack of staff, drugs, or medical equipment in the hospital (28.9%), followed by unexpected patient death or complications (19.7%). Half of the events (50.3%) were not reported primarily due to the lack of a reporting system in the hospital or health center.

**TABLE 2:** The frequency and pattern of WPV reported by HCWs during the COVID‐19 pandemic in Sudan, 2021

Variables	*n*	(%)
Frequency of violence attacks (*n* = 620)		
Once	43	(6.9)
Two‐three times	169	(27.3)
More than three times	408	(65.8)
Type of violence^a^		
Verbal (swearing or screaming)	568	(91.6)
Physical	310	(50.0)
Sexual harassment	68	(11.0)
Other	1	(0.2)
Place of violent attacks (*n* = 620)		
Emergency room	291	(46.9)
Outpatient clinic	136	(21.9)
Pharmacy	81	(13.1)
Intensive care unit	53	(8.6)
Wards	46	(7.4)
Others	13	(2.1)
Offender identity^a^		
Patient	313	(50.5)
Patient's family	545	(87.9)
Friends	221	(35.6)
Others	6	(1.0)
Trigger for violence (*n* = 620)		
Lack of staff, drugs, or medical equipment in the hospital	179	(28.9)
Unexpected patient death or complications	122	(19.7)
Unexpected bill	113	(18.2)
No explanation about the patient's condition to him or his family	110	(17.7)
Health worker's rude behavior	78	(12.6)
Others	18	(2.9)
Violent events were reported (*n* = 620)		
Yes	308	(49.7)
No	312	(50.3)
The main reason for not reporting (*n* = 312)		
Lack of reporting system in the hospital	178	(57.0)
No actions will be taken against offenders	97	(31.1)
Their behavior was reasonable due to many reasons	23	(7.4)
It is part of the job, and I have to tolerate it	10	(3.2)
Others	4	(1.3)

^a^Multiple responses allowed.

Results of the unadjusted analysis of factors associated with suffering WPV during the COVID‐19 pandemic are presented in Table 3. The demographic factors that were significantly associated with exposure to violence were participants’ occupation (*p* < 0.001), age (*p* = 0.001), marital status (*p* = 0.002), and years of working experience (*p* = 0.020). However, there was no significant association between exposure to violence and HCWs’ sex (*p* = 0.548) or shift time worked (*p* = 0.059).

**TABLE 3:** Factors associated with suffering WPV among HCWs in Sudan during the COVID‐19 pandemic, 2021

	Experienced workplace violence					
	Yes (*n* = 620)		No (*n* = 172)			
Variables	*n*	(%)	*n*	(%)	*χ* 2	*p*‐Value
Occupation						
Physician	281	(45.3)	67	(39.0)	53.104	<0.001
Nurse	141	(22.7)	18	(10.5)		
Dentist	63	(10.2)	22	(12.8)		
Pharmacist	94	(15.2)	30	(17.4)		
Laboratory technician	37	(6.0)	21	(12.2)		
Others	4	(0.6)	14	(8.1)		
Shift time worked						
Day shift	354	(57.1)	112	(65.1)	3.576	0.059
Night shift	266	(42.9)	60	(34.9)		
Gender						
Male	276	(44.5)	81	(47.1)	0.361	0.548
Female	344	(55.5)	91	(52.9)		
Age (years)						
<30	257	(41.5)	99	(57.6)	14.418	<0.001
30– < 40	203	(32.7)	38	(22.1)		
≥40	160	(25.8)	35	(20.3)		
Marital status						
Married	303	(48.9)	63	(36.6)	12.212	0.002
Single	295	(47.6)	95	(55.2)		
Divorced/Widow	22	(3.5)	14	(8.1)		
Years of experience						
<5 years	283	(45.7)	99	(57.6)	7.851	0.020
5–10 years	172	(27.7)	35	(20.3)		
More than 10 years	165	(26.6)	38	(22.1)		

*χ* 2: Chi‐square test.

## DISCUSSION

High proportions of HCWs in Sudan had experienced WPV during the COVID‐19 pandemic. The current study findings point to the catastrophic and complicated case of WPV against HCWs in Sudan, which calls for immediate and radical interventions. A previous study of WPV among physicians in Khartoum state in Sudan reported a 50% magnitude of WPV during 2019–2020 [14]. Accordingly, the current findings suggest that the COVID‐19 pandemic has aggravated the frequency of WPV in Sudan. This result can be compared with a study in Iraq in which 87.3% of physicians experienced hospital violence [20]. Also, studies from Jordan, Brazil, and China reported that 65.5%, 47.6%, and 20.4% of HCWs were exposed to WPV, respectively [5, 27, 28]. Verbal abuse was the most common (91.6%) type of violence encountered by the current study respondents. This was the case in China, as 94.1% of frontline clinicians suffered verbal abuse during the pandemic [29]. Overall, these findings prompt the need for contextually applicable measures, as implementing a uniform solution that suits all is unlikely to be effective.

The emergency department had the largest percentage of violent events (46.9%). This finding is consistent with previous studies of WPV in Sudan [14]. This could be explained by heightened emotional tension because of the situation of the patients, and thus, the relatives and patients are more emotionally volatile. In addition to the emotionally charged, overworked and stressed workers in emergency settings [16, 30]. Moreover, this result could also be related to political protests in Sudan during the epidemic, where hospitals’ emergency departments and HCWs were frequently attacked by security forces [31]. Similarly to this result, the likelihood of violence was higher among emergency department workers in both China and Ethiopia [29, 32]. The current study found that patients' families were the most violent offenders. The same was noted in studies from Egypt, Palestine, and India [21, 33, 34]. The lack of staff, drugs, or medical equipment in the hospital was the most common perceived trigger for violence in the current study. Similarly, multiple shortcomings of the health services were the triggers of violence against physicians treating COVID‐19 patients in Peru [35]. This result is significant because knowing the reason and implementing a comprehensive health and safety preventive strategy are the keys to understanding, preventing, and dealing with WPV. The multiple gaps and deficiencies seen in hospitals during the pandemic should be a lesson to create a necessary protocol and increase workplace safety in favor of medical personnel for the future.

Our findings showed that half of the attacks were not reported primarily due to the lack of reporting systems. Underreporting of violent attacks is widely documented in the published literature on WPV. This issue exists for many reasons; many HCWs consider violence a part of the job, and some believe that no action will be taken against perpetrators, in addition to the lack of awareness about policies and reporting systems [36]. In a study among Chinese nurses, half of the participants were unaware of how and what types of violence to report, and half believed that the hospital paid greater attention to patients rather than the staff [37]. Another study showed that only less than a third of HCWs who experienced verbal and physical violence had reported the incidents to the hospital authority, with more than half believing that no action was taken against the offenders [6]. This was also true among nurses in Ethiopia, where more than half confirmed the lack of reporting procedures [32].

According to the current study findings, age, marital status, years of work experience, and occupation were strongly associated with exposure to violence. When dealing with WPV, these factors play a significant role in influencing subsequent responses. For example, older HCWs with more experience would expect to have better communication skills and more training and practice of de‐escalation strategies that emphasize improvements in behavioral responses in the case of an assault. A study of Chinese frontline HCWs during the COVID‐19 epidemic found that WPV was significantly associated with participants' sociodemographic factors [29]. In the current study, however, there was no significant relationship between exposure to violence and HCWs' gender or time shift worked. This finding magnifies the extent of the problem, both genders might suffer WPV at any time, which could be due to the increased frequency of violence each day in Sudan amid political protests and conflict. Similarly, there was no difference in reports of violence between male and female doctors during the pandemic in Iraq [20]. Though, there are mixed findings on the relationship of sex/gender with WPV during the COVID‐19 pandemic. For example, a study conducted among Peruvian HCWs has demonstrated that the female gender was significantly associated with increased odds of suffering violence and aggression [35]. In contrast, the male gender was a significant predictor of suffering WPV during the COVID‐19 pandemic among Jordanian and Chinese HCWs [5, 29].

### Limitations

This study encountered several limitations; despite efforts to adjust for sampling bias, the use of the non‐probability convenience sampling in recruiting participants limits the study findings' generalizability to all HCWs in Sudan. In addition, using an online‐based questionnaire for data collection poses a risk of selection bias favoring only HCWs with active access to the internet. Since the present study further assessed previous experience with violent events, the results are prone to recall bias. Finally, we used a self‐report questionnaire for data collection, hence our findings might have been affected by social desirability bias.

## CONCLUSIONS

WPV was rampant among the HCWs in Sudan during the COVID‐19 pandemic. The most agreed upon triggers of WPV in the present study were lack of staff, drugs, or medical equipment in the hospital, and unexpected patient death or complications. Participants’ age, marital status, years of working experience, and occupation were significantly associated with suffering WPV. The current findings are presented to draw the attention of policy leaders and stakeholders to this alarming problem in Sudan and prompt the need for policy and system interventions.

## AUTHOR CONTRIBUTIONS

Yasir Elhadi: Conceptualization; Data curation (lead); Formal analysis; Methodology; Project administration; Resources; Writing ‐ original draft; Writing ‐ review & editing (lead). Hammad Mohamed, Abdelmuniem Ahmed, Islam Haroun, Mohamed Hag, Ehssan Farouk, Moaaz Almadani, Alanood Mohamed, Mohammed Adam, Osman Abdelhamed, Elhadi Salih and Sahar Mohamed: Data curation; Writing ‐ review & editing. Mohammed Sanosi: Data curation; Supervision; Writing ‐ review & editing.

## CONFLICT OF INTEREST

Elhadi Y.A.M is a Youth Editorial Board member of Public Health Challenges and co‐author of this article. He was excluded from editorial decision‐making related to the acceptance of this article for publication in the journal.

## ETHICAL APPROVAL STATEMENT

This study has been approved by the Health Sector Ethical Review Committee, Faculty of Medicine, University of Gezira in Sudan.

## Data Availability

The data that support the findings of this study are available from the corresponding author upon reasonable request.

## References

[1] Nagpal N , Nagpal N , Kataria N , Parikh P . Violence against health care professionals and facilities‐local insights about a global malady. South Asian J Cancer. 2020; 9(4): 257‐260. 10.1055/S-0041-1726137 34131577 PMC8197649

[2] Alhamad R , Suleiman A , Bsisu I , et al. Violence against physicians in Jordan: an analytical cross‐sectional study. PLoS One. 2021; 16(1). 10.1371/JOURNAL.PONE.0245192 PMC7833172 33493170

[3] Occupational Safety and Health Administration (OSHA) . Guidelines for preventing workplace violence for health care and social service workers. Prairie Rose. 1997;66(1). 9326080

[4] Liu J , Gan Y , Jiang H , et al. Prevalence of workplace violence against healthcare workers: a systematic review and meta‐analysis. Occup Environ Med. 2019; 76(12): 927‐937. 10.1136/OEMED-2019-105849 31611310

[5] Ghareeb NS , El‐Shafei DA , Eladl AM . Workplace violence among healthcare workers during COVID‐19 pandemic in a Jordanian governmental hospital: the tip of the iceberg. Environ Sci Pollut Res Int. 2021; 28(43): 61441‐61449. 10.1007/S11356-021-15112-W 34173953 PMC8233595

[6] Abdellah RF , Salama KM . Prevalence and risk factors of workplace violence against health care workers in emergency department in Ismailia, Egypt. Pan Afr Med J. 2017; 26. 10.11604/PAMJ.2017.26.21.10837 PMC5398248 28451000

[7] Fallahi‐Khoshknab M , Oskouie F , Najafi F , Ghazanfari N , Tamizi Z , Afshani S . Physical violence against health care workers: a nationwide study from Iran. Iran J Nurs Midwifery Res. 2016; 21(3): 232. 10.4103/1735-9066.180387 27186199 PMC4857656

[8] Devi S . COVID‐19 exacerbates violence against health workers. Lancet. 2020; 396(10252): 658. 10.1016/S0140-6736(20)31858-4 32891198 PMC7470723

[9] International Committee of the Red Cross . ICRC: 600 Violent Incidents against Health Workers . Accessed March 2, 2022. https://www.icrc.org/en/document/icrc‐600‐violent‐incidents‐recorded‐against‐healthcare‐providers‐patients‐due‐covid‐19

[10] National Nurses United . National Nurse Survey Exposes Hospitals’ Knowing Failure to Prepare for a Covid‐19 Surge during Flu Season. 2020. Accessed March 2, 2022. https://www.nationalnursesunited.org/press/national‐nurse‐survey‐4‐exposes‐hospitals‐knowing‐failure‐prepare‐covid‐19‐surge

[11] Bhatti OA , Rauf H , Aziz N , Martins RS , Khan JA . Violence against healthcare workers during the COVID‐19 pandemic: a review of incidents from a lower‐middle‐income country. Ann Glob Heal. 2021; 87(1). 10.5334/AOGH.3203 PMC8064297 33977084

[12] El‐Sadig SM , Fahal LA , Abdelrahim ZB , Ahmed ES , Mohamed NS , Siddig EE . Impact of COVID‐19 on doctors and healthcare providers during the pandemic in Sudan. Trans R Soc Trop Med Hyg. 2021; 0: 1‐2. 10.1093/trstmh/trab016 PMC7928645 33547896

[13] Sudan Doctors Welcome New Law That Protects Them 2020. Accessed March 2, 2022. https://www.dabangasudan.org/en/all‐news/article/sudan‐doctors‐welcome‐new‐law‐that‐protects‐them

[14] Mohamed Elamin M , Boushra Hamza S , Abbasher K , et al. Workplace violence against doctors in Khartoum state, Sudan, 2020. Sudan J Med Sci. 2021; 16(2): 301‐319‐301–319. 10.18502/SJMS.V16I2.9296

[15] Watson A , Jafari M , Seifi A . The persistent pandemic of violence against health care workers. Am J Manag Care. 2020; 26(12): E377‐E379. 10.37765/AJMC.2020.88543 33315330

[16] Berlanda S , Pedrazza M , Fraizzoli M , De Cordova F . Addressing risks of violence against healthcare staff in emergency departments: the effects of job satisfaction and attachment style. Biomed Res Int. 2019; 2019. 10.1155/2019/5430870 PMC6558649 31275976

[17] Zafar W , Khan UR , Siddiqui SA , Jamali S , Razzak JA . Workplace violence and self‐reported psychological health: coping with post‐traumatic stress, mental distress, and burnout among physicians working in the emergency departments compared to other specialties in Pakistan. J Emerg Med. 2016; 50(1): 167‐177.e1. 10.1016/J.JEMERMED.2015.02.049 26412103

[18] Lim MC , Jeffree MS , Saupin SS , Giloi N , Lukman KA . Workplace violence in healthcare settings: the risk factors, implications and collaborative preventive measures. Ann Med Surg. 2022; 78: 103727. 10.1016/J.AMSU.2022.103727 PMC9206999 35734684

[19] Patil P , Taneja S . Medicine or martyrdom? A peek into the rising violence against doctors during times of COVID 19. J Fam Med Prim Care. 2021; 10(8): 2732. 10.4103/JFMPC.JFMPC_1790_20 PMC8483096 34660396

[20] Lafta R , Qusay N , Mary M , Burnham G . Violence against doctors in Iraq during the time of COVID‐19. PLoS One. 2021; 16(8). 10.1371/JOURNAL.PONE.0254401 PMC8345879 34358239

[21] Arafa A , Shehata A , Youssef M , Senosy S , Violence against healthcare workers during the COVID‐19 pandemic: a cross‐sectional study from Egypt. Arch Environ Occup Health. Published online 2021. 10.1080/19338244.2021.1982854 34590540

[22] Gaafar N , Hemmeda L , Violence against healthcare workers in haj el‐safi teaching hospital, Sudan: a cross‐sectional study. Published online July 5, 2022. doi:10.21203/RS.3.RS-1815105/V1

[23] von Elm E , Altman DG , Egger M , Pocock SJ , Gøtzsche PC , Vandenbroucke JP . The strengthening the reporting of observational studies in epidemiology (STROBE) statement: guidelines for reporting observational studies. J Clin Epidemiol. 2008; 61(4): 344‐349. 10.1016/J.JCLINEPI.2007.11.008 18313558

[24] Alfuqaha OA , Albawati NM , Alhiary SS , et al. Workplace violence among healthcare providers during the COVID‐19 health emergency: a cross‐sectional study. Behav Sci. 2022; 12(4): 106. 10.3390/BS12040106 35447678 PMC9026762

[25] Byon HD , Sagherian K et al., Nurses’ experience with type II workplace violence and underreporting during the COVID‐19 pandemic. Published online August 3, 2021. 10.1177/21650799211031233 34344236

[26] Alsaleem SA , Alsabaani A , Alamri RS , et al. Violence towards healthcare workers: a study conducted in Abha City, Saudi Arabia. J Family Community Med. 2018; 25(3): 188. 10.4103/JFCM.JFCM_170_17 30220849 PMC6130164

[27] Bitencourt MR , Alarcão ACJ , Silva LL , et al. Predictors of violence against health professionals during the COVID‐19 pandemic in Brazil: a cross‐sectional study. PLoS One. 2021; 16(6): e0253398. 10.1371/JOURNAL.PONE.0253398 34138953 PMC8211185

[28] Wang W , Lu L , Kelifa MM , et al. Mental health problems in chinese healthcare workers exposed to workplace violence during the COVID‐19 outbreak: a cross‐sectional study using propensity score matching analysis. Risk Manag Healthc Policy. 2020; 13: 2827. 10.2147/RMHP.S279170 33299370 PMC7721299

[29] Yang Y , Li Y , An Y , et al. Workplace violence against chinese frontline clinicians during the COVID‐19 pandemic and its associations with demographic and clinical characteristics and quality of life: a structural equation modeling investigation. Front Psychiatry. 2021;12:414. 10.3389/FPSYT.2021.649989/BIBTEX PMC8081975 33935836

[30] Isbell LM , Boudreaux ED , Chimowitz H , Liu G , Cyr E , Kimball E . What do emergency department physicians and nurses feel? A qualitative study of emotions, triggers, regulation strategies, and effects on patient care. BMJ Qual Saf. 2020; 29(10): 1. 10.1136/BMJQS-2019-010179 PMC7363518 31941799

[31] Osman M , Alassam MN . Military attacks on health workers in Sudan. Lancet. 2022; 0(0). 10.1016/S0140-6736(22)00319-1 35189075

[32] Fute M , Mengesha ZB , Wakgari N , Tessema GA . High prevalence of workplace violence among nurses working at public health facilities in Southern Ethiopia. BMC Nurs. 2015; 14(1): 1. 10.1186/S12912-015-0062-1 25767412 PMC4357058

[33] Kumar M , Verma M , Das T , Pardeshi G , Kishore J , Padmanandan A . A study of workplace violence experienced by doctors and associated risk factors in a tertiary care hospital of South Delhi, India. J Clin Diagn Res. 2016; 10(11): LC06. 10.7860/JCDR/2016/22306.8895 PMC5198359 28050406

[34] Hamdan M , Abu Hamra A . Workplace violence towards workers in the emergency departments of Palestinian hospitals: a cross‐sectional study. Hum Resour Health. 2015; 13(1). 10.1186/S12960-015-0018-2 PMC4435901 25948058

[35] Muñoz del Carpio‐Toia A , Begazo Muñoz del Carpio L , Mayta‐Tristan P , Alarcón‐Yaquetto DE , Málaga G . Workplace violence against physicians treating COVID‐19 patients in peru: a cross‐sectional study. Jt Comm J Qual Patient Saf. 2021; 47(10): 637. 10.1016/J.JCJQ.2021.06.002 34257040 PMC8200256

[36] Phillips JP . Workplace violence against health care workers in the United States. N Engl J Med. 2016; 374(17): 1661‐1669. 10.1056/NEJMRA1501998/SUPPL_FILE/NEJMRA1501998_DISCLOSURES.PDF 27119238

[37] Song C , Wang G , Wu H . Frequency and barriers of reporting workplace violence in nurses: an online survey in China. Int J Nurs Sci. 2021; 8(1): 65‐70. 10.1016/J.IJNSS.2020.11.006 33575447 PMC7859538

